# No causal genetic relationships between atrial fibrillation and vascular dementia: A bidirectional Mendelian randomization study

**DOI:** 10.3389/fcvm.2023.1071574

**Published:** 2023-06-30

**Authors:** Ya-fang Gao, Tian-yu Jin, Yan Chen, Ya-hui Ding

**Affiliations:** ^1^ Department of Cardiology, Zhangzhou Affiliated Hospital of Fujian Medical University, Zhangzhou, China; ^2^ Heart Center, Department of Cardiovascular Medicine, Zhejiang Provincial People's Hospital (Affiliated People's Hospital, Hangzhou Medical College), Hangzhou, China; ^3^ Department of Rehabilitation Medicine, Wenzhou Medical University, Wenzhou, China

**Keywords:** atrial fibrillation, vascular dementia, causality, Mendelian randomization, risk

## Abstract

**Background:**

Numerous observational studies have suggested that atrial fibrillation (AF) was associated with an increased risk of vascular dementia (VaD). However, the causal genetic relationships between AF and VaD remains unclear. To evaluate the effect of AF on VaD, we performed the Mendelian randomization (MR) analysis to investigate the causal genetic relationships between AF and VaD.

**Methods:**

The bidirectional MR analysis was conducted to explore the causal relationships between exposure and disease. We applied a series of quality assessments to select significantly and independently single nucleotide polymorphisms (SNPs) from publicly available large-scale genome-wide association studies (GWAS) databases. Three methods [Inverse variance weighted method (IVW), MR-Egger method, and weighted median (WM)method] were used to derive MR estimates. In order to ensure reliable MR results, sensitivity analyses were performed to evaluate the horizontal pleiotropy and heterogeneity.

**Results:**

Our MR analyses revealed no significant genetic relationships between AF and the risk of VaD (IVW: OR =  1.10, 95%CI = 0.95–1.28, *P *= 0.20). In the reverse direction analysis, there was no evidence to support a significant genetic relationship of VaD with AF risk (IVW: OR =  1.00, 95% CI = 0.99–1.01, *P *= 0.52). Consistent results were obtained using different MR methods. Sensitivity analyses suggested no significant horizontal pleiotropy and heterogeneity in the study.

**Conclusion:**

This MR analysis did not provide evidence to support the causal genetic relationships between AF on VaD risk and the causal effect of VaD on AF risk.

## Introduction

Vascular dementia (VaD), accounting for approximately 15% of dementia cases, is the second most common dementia after Alzheimer's disease (AD) (1). It is described as a brain injury due to impaired cerebral blood flow resulting in severe cognitive decline that interferes with family, occupational and social life (2). Similar to AD, the incidence of VaD increases rapidly with advancing age (3). As the global average life expectancy increases, the prevalence of dementia in the aging population undoubtedly imposes an enormous burden on families, healthcare institutions and the government. Currently, there are no effective and licensed treatments for VaD (1). Numerous epidemiological studies have found that the incidence of dementia has declined in recent years because of reductions in vascular risk factors and improvements in treatment modalities (4–6). Therefore, the risk factors for VaD have received much attention recently, such as advanced age, hypertension, diabetes, obesity, and cardiovascular factors (7). However, some of the risk factors remain controversial.

Atrial fibrillation (AF) is a common cardiac arrhythmia with a high rate of disability and mortality (8). It is usually associated with multiple comorbidities (9). There were approximately 37.6 million cases of AF worldwide in 2017, and the number is increasing yearly (10). Patients with AF appear to be more susceptible to VaD and exhibit a worse prognosis. A meta-analysis showed that AF was significantly associated with an increased risk of VaD [odds ratio (OR) = 1.7, 95% confidence interval (CI): 1.4–3.5] (11). In the meta-analysis, we found that current cohort studies were usually corrected for risk factors such as age, hypertension, coronary artery disease, diabetes, and hyperlipidemia. However, there is always a risk of unmeasured confounders, which is relevant to address as well as the advantage of MR studies when it comes to causality.

Mendelian randomization (MR) is a robust statistical method that employs genetic variables as instruments to reveal genetic causality between exposure and disease (12). MR analysis enables to overcome reverse causality relationships as genes are essentially stable after fertilization (13). Moreover, MR analysis can eliminate the interference of confounding factors owing to the random assignment of alleles (14).

In the present study, we used the publicly available database of large-scale genome-wide relationships studies (GWAS) and applied a two-sample bidirectional MR analysis to investigate the potential causal relationships between AF and VaD.

## Methods

### Ethics statement

The study was conducted in accordance with the Declaration of Helsinki. Summary-level statistics from the FinnGen GWAS database for VaD are publicly available (URL: http://r5.risteys.finngen.fi/phenocode/F5_VASCDEM, last accessed on 16 August 2022). The summary statistics for the AF are provided in the GWAS meta-analysis (URL:http://www.nature.com/articles/s41588-018-0171-3, last accessed on 16 August 2022). As the study examined anonymized and summary-level data, the informed consent was waived.

### Study design

**Figure 1:**
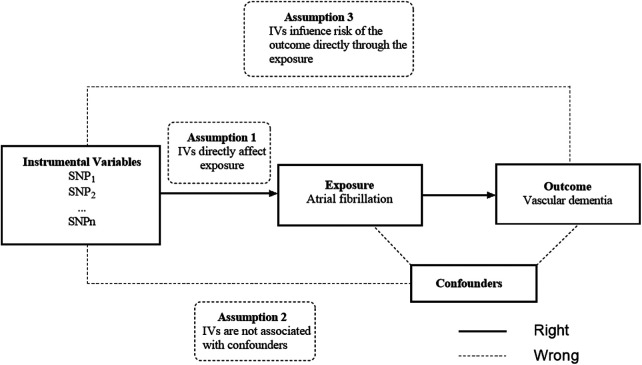
An overview of the study design.

**Figure 2:**
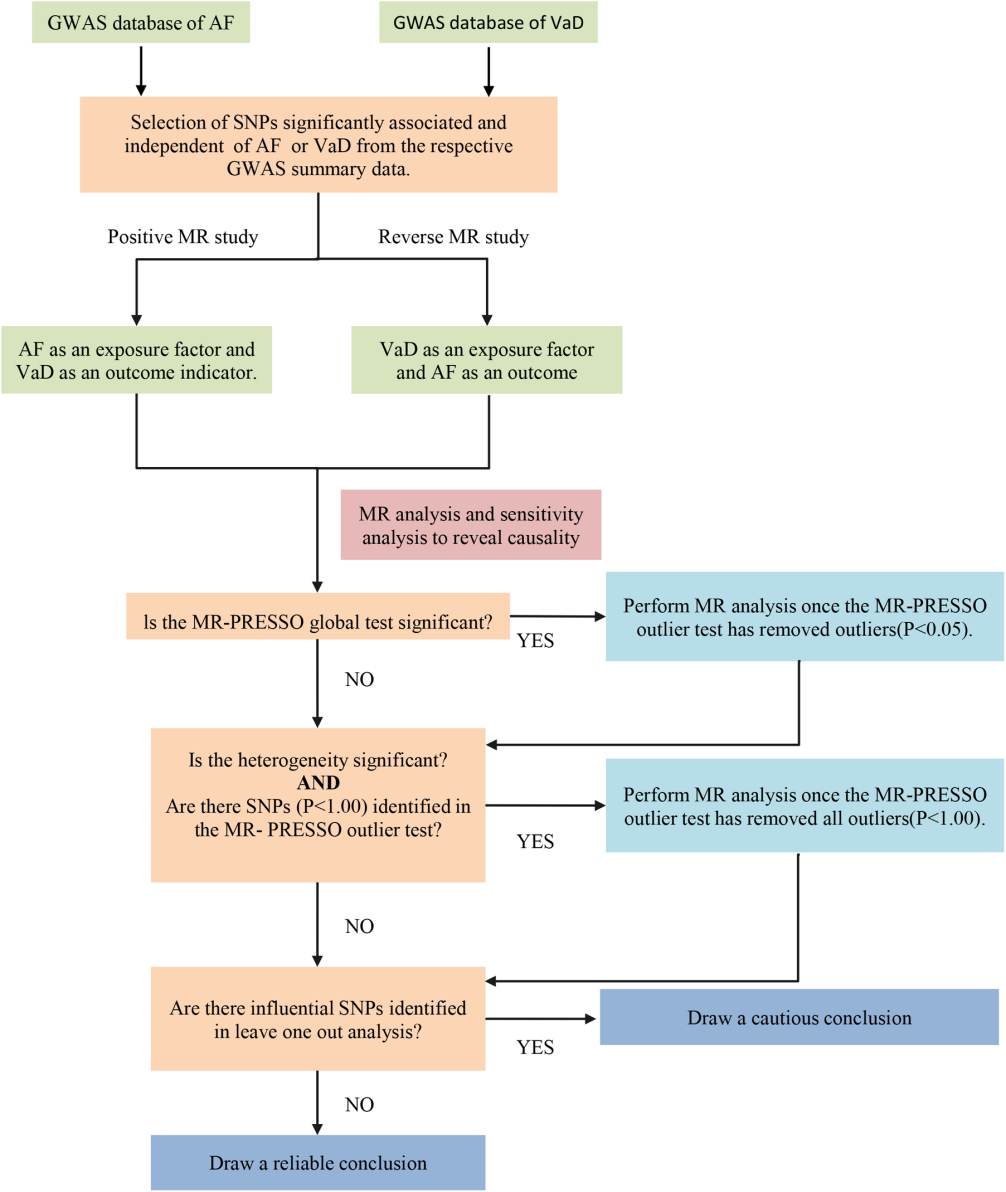
The flow chart of the bidirectional MR process. AF, atrial fibrillation; VaD, vascular dementia; SNP, single nucleotide polymorphisms; MR-PRESSO, MR-pleiotropy residual sum and outlier.

We conducted a two-sample MR study to explore the causal relationship between AF and VaD risk using the largest publicly available GWAS database. The MR study was performed based on the hypothesis that genetic variants affect the outcome only through exposure rather than other pathways. The selected IVs should be independent of confounding factors (Figure 1) (15). The bidirectional MR analysis flow is shown in Figure 2.

### GWAS meta-analysis for AF

For the exposure data, we selected the largest publicly summarized GWAS database of AF. In total, more than 1,000,000 participants of European ancestry were enrolled, including 60,620 cases and 970,216 controls (16). This database compares data from six cohort studies (The Nord-Trøndelag Health Study (HUNT), the Michigan Genomics Initiative (MGI), deCODE, DiscovEHR, AFGen Consortium, and the UK Biobank). The diagnostic criteria for AF were the International Classification of Diseases (ICD) 9 and ICD 10 code. SNPs information of effect allele (EA), effect frequency (EAF), effect size (*β*) and *P* value were extracted. The baseline characteristics of the database are shown in Table 1.

**Table 2:** The Mendelian randomization of Atrial fibrillation and Vascular dementia.

Exposure	Outcome	SNPs	Method	OR	95% CI	*P* value	Q_*P* value	Intercept (*P* value)
Atrial fibrillation	Vascular dementia	99	IVW	1.10	0.95–1.28	0.20	0.19	−0.0087 (0.48)
			MR-Egger	1.21	0.90–1.62	0.21	0.39	
			WM	1.18	0.90–1.55	0.24		
Vascular dementia	Atrial fibrillation	12	IVW	1.00	0.99–1.01	0.52	0.45	0.0019 (0.61)
			MR-Egger	1.00	0.99–1.01	0.93	0.18	
			WM	1.01	0.99–1.02	0.38		

SNPs, single nucleotide polymorphisms; OR, odds ratio; CI, confidence interval; IVW, inverse-variance weighted; WM, weight median.

### GWAS database for VaD

As for the outcome data, we obtained pooled-level data from the Finn consortium for 881 cases and 211,508 controls in the European population. All cases of VaD were diagnosed according to the ICD 10 code.

### Methods for genetic instrumental variable selection

**Figure 3:**
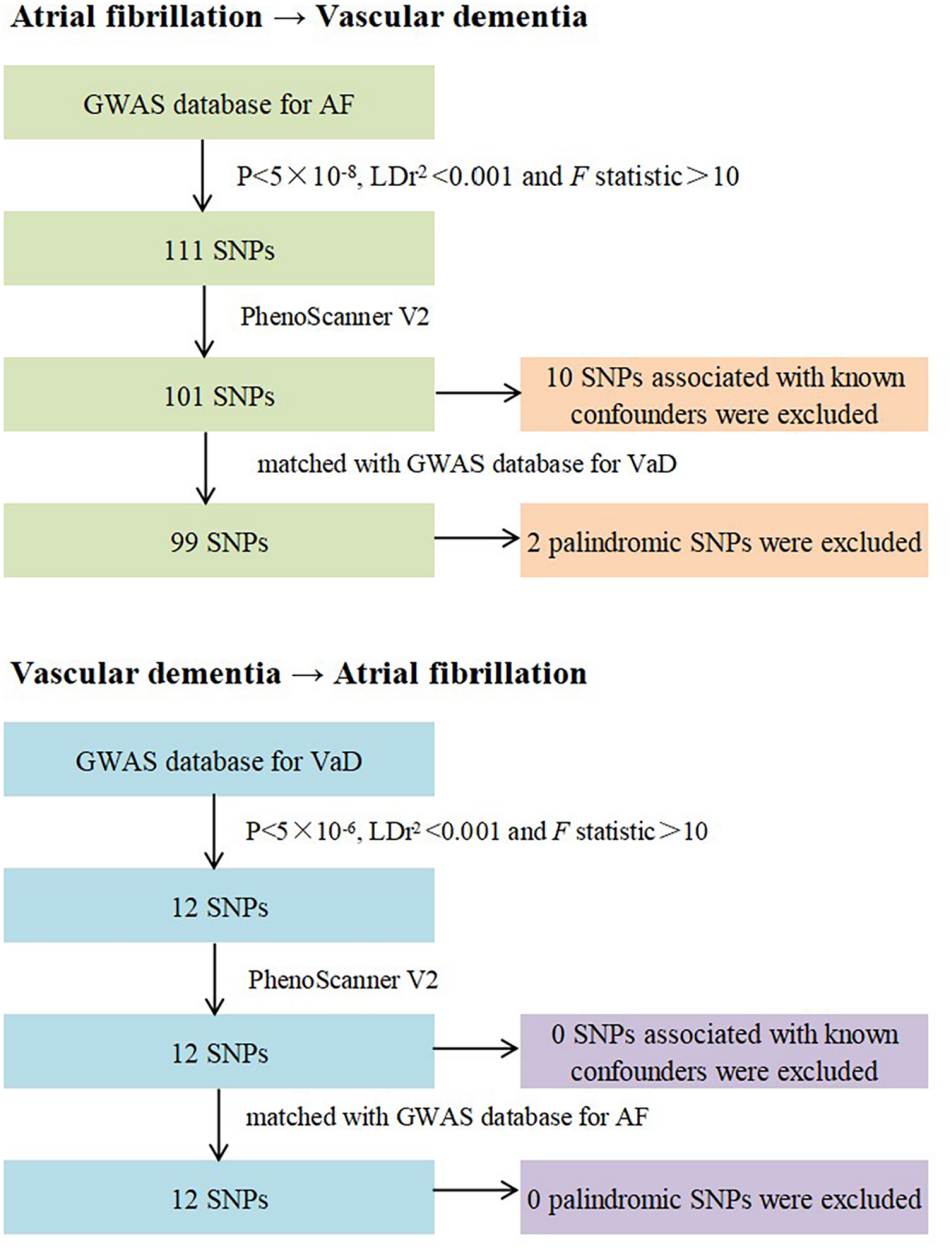
Flow chart of the performed analysis and construction of the genetic instruments.

Figure 3 shows the Flow chart of the performed analysis and construction of the genetic instruments. Firstly, each SNP should be associated closely with AF. SNPs were included if they reached genome-wide significance (*P* < 5 × 10^−8^). SNPs with F statistic great 10 to avoid bias caused by weak IVs. The formula for F statistic is detailed in Supplementary Material S1 (17). Secondly, each SNP should not be associated with confounders of the exposure-outcome relationship. SNPs were included if they satisfied the independence [linkage disequilibrium (LD), r^2 ^≤ 0.001]. Thirdly, each SNP should only affect the risk of outcome indicators through exposure factors and no other pathways (no pleiotropy). We remove the SNPs associated with confounding elements of VaD using PhenoScanner V2 (www.phenoscanner.medschl.cam.ac.uk) (18).

We extracted 111 significant and independent SNPs associated with AF from the largest GWAS database. 10 SNPs (rs284277, rs6689306, rs2540949, rs1458038, rs1838747, rs56201652, rs11191116, rs7915134, rs12245149, rs2759301) were removed using PhenoScanner V2 as these SNPs were associated with known confounders (hypertension, coronary artery atherosclerotic heart disease, body mass index and education), and 2 SNPs were excluded due to their mutual pairing in the double helix structure. The characteristics of the final SNPs for AF are shown in Supplementary Table S1.

Since setting the threshold of *P* value at <5 × 10^−8^ obtains few SNPs for VaD, we relaxed the threshold of *P* value to <5 × 10^−6^ (19, 20). 12 SNPs were obtained from the GWAS database (Supplementary Table S2). No SNPs were included because of palindromic sequence and PhenoScanner V2.

All included SNPs F statistics were greater than 10, indicating no weak instrument bias in our study.

### Methods for summary-level MR

We used three approaches to evaluate MR estimates of AF with VaD, including inverse-variance weighted (IVW), weight median (WM) and MR-Egger. The IVW method was the primary method as it was the Wald ratio of individual SNPs for meta-analysis, which assumes that all IVs were valid (21). The intercept restriction of the IVW method is zero, which indicates that it can provide unbiased estimates if there is no pleiotropy (22). In the MR study, the presence of pleiotropy may lead to bias and unstable MR estimates (23). Therefore, we applied MR-Egger and WM as additional MR estimates. When up to 50% of the information comes from invalid instrumental variables, the WM method can be used as more reliable causal MR estimates (24). Although the MR-Egger approach is known to be somewhat resistant to the existence of pleiotropy, it nevertheless suffers from its effects and has reduced statistical power (25). However, it can detect whether the pleiotropy was present (22). Although these methods were less accurate, they might provide wider confidence intervals (CIs) (26). If these methods yield inconsistent MR estimates, we will tighten the IVs and reperform the analysis.

### Sensitivity analysis for summary-level MR

Sensitivity analyses were applied to ensure the reliability of the MR results. The heterogeneity of each SNP was evaluated by Cochran's Q statistic. In the present study, we used Cochran's Q statistic (*P *< 0.05) to represent the significant heterogeneity between the estimates of each included SNP. The intercept of the MR-Egger regression provided an indication of horizontal pleiotropy (*P *< 0.05 indicates the existence of horizontal pleiotropy). In addition, MR-Pleiotropy Residual Sum and Outlier methods (MR-PRESSO) were used to detect the outliers and potential horizontal pleiotropy (global *P *< 0.05 suggests the presence of horizontal pleiotropy). If outliers are found, they will be removed to obtain a more accurate corrected estimate (27). Finally, leave-one-out analysis was employed to evaluate the stability of MR estimates after excluding the individual SNP (28). Additionally, we switched the outcome and exposure and reperformed the MR and sensitivity analyses to avoid the possibility of reverse causation. The two-sample MR study was analyzed by Two-Sample MR (version 0.5.5) in R (version 4.2.0).

## Results

### MR estimates and sensitivity analyses

**Figure 4:**
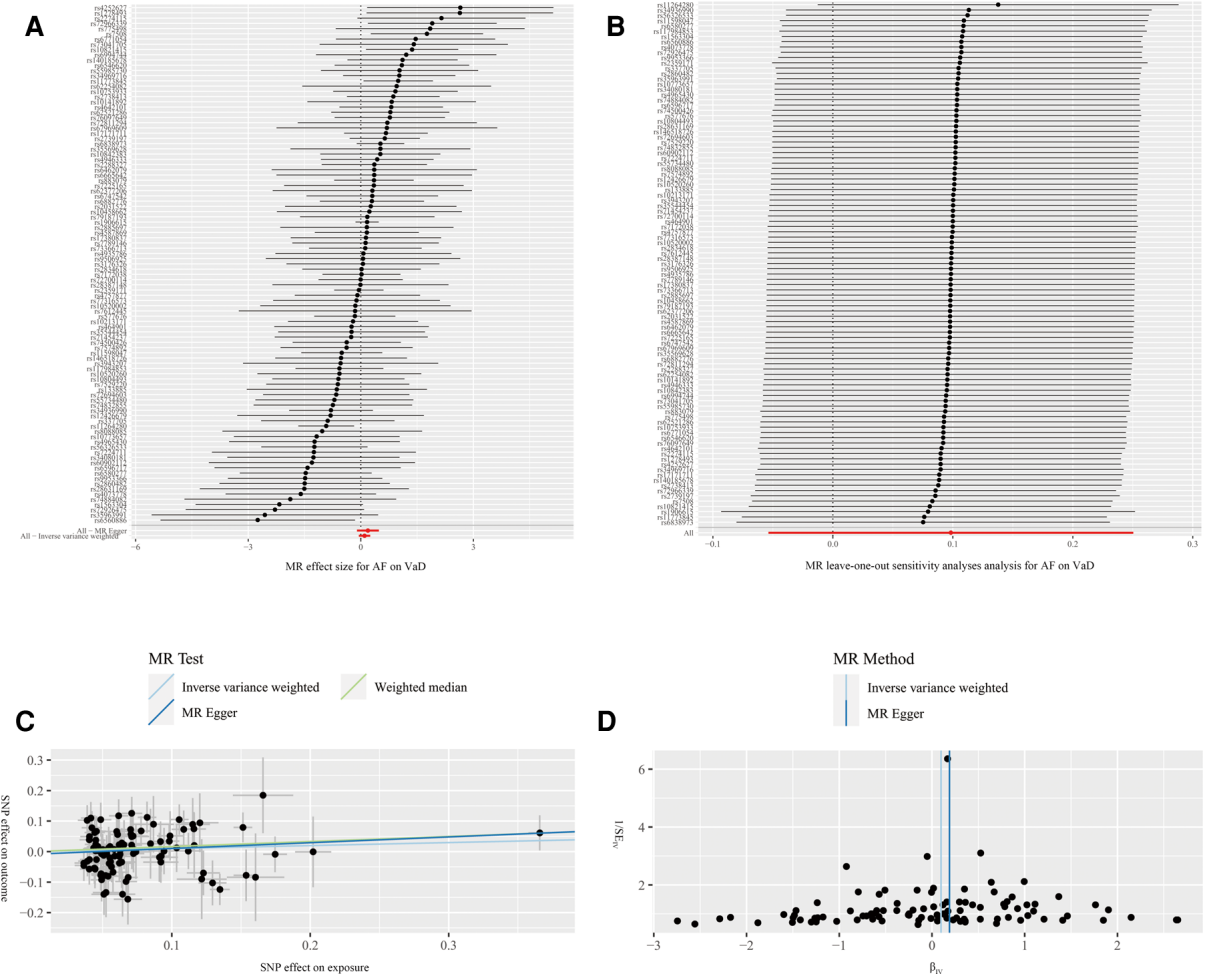
Forest plot (**A**), leave-one-out analysis (**B**), scatter plot (**C**) and funnel plot (**D**) of the effect of atrial fibrillation on vascular dementia (VaD). Figure **A** shows the MR estimate of each SNP effect on VaD. Figure **B** depicts the changes of MR estimates after excluding individual SNP. The three lines in Figure **C** illustrate the estimated effect sizes by three MR methods (IVW, MR-Egger and WM). No significant horizontal pleiotropy was found for the MR-Egger's intercept. Figure **D** demonstrates that the funnel plot is symmetric, which indicates that the MR estimates are reliable.

The results of MR estimation and sensitivity analyses are presented in Table 2. According to the IVW result, no significant causal relationship between AF and the risk of VaD was found (OR = 1.10, 95% CI* *= 0.95–1.28, *P* = 0.20). The MR-Egger (OR = 1.21, 95% CI* *= 0.90–1.62, *P* = 0.21) and WM (OR = 1.18, 95% CI* *= 0.90–1.55, *P* = 0.24) methods showed similar results (Figure 4A). Cochran's Q statistic showed no significant heterogeneity in the estimates of included SNPs (*P* = 0.19). In addition, the leave-one-out analysis also suggested that the result of MR estimates was stable (Figure 4B). The MR Egger's intercept (intercept = −0.01 and *P *= 0.48) and MR-PRESSO (global *P* = 0.18) revealed no significant horizontal pleiotropy in our study (Figure 4C). Further, the funnel plot was symmetrical, indicating that the results were reliable (Figure 4D).

**Table 1:** Baseline information from the GAWS database for AF.

Characteristics	HUNT	deCODE*	MGI	DiscoverEHR	UKB	AFGen**
Cases, *N*	6493	13,471	1226	6679	14,820	17,931
Controls, *N*	63,142	358,161	11,049	41,803	380,919	115,142
Subjects, N	69,635	371,632	12,275	48,482	395,739	133,073
Unique markers tested, *N*	20,013,723	18,134,320	12,948,440	12,184,179	28,226,282	11,792,062
Sex, *N* women (%)	36,887 (53)	181,792 (49)	6,558 (53)	29,530 (61)	214,658 (54)	**
**Age at first AF diagnosis, median (IQR)**						
Cases	76 (67–83)	74 (65–82)	65 (50–80)	/	/	**
**Hypertension, *N* (%)**						
Cases	5,465 (84)	7,444 (55)	962 (78)	5,896 (88)	11,019 (74)	**
Controls	30,117 (48)	46,828 (13)	4,172 (38)	21,799 (52)	183,837 (48)	**
**Type 2 diabetes, *N* (%)**						
Cases	1,231 (19)	1,754 (13)	408 (33)	2,214 (33)	2,334 (16)	**
Controls	4,151 (7)	9,549 (3)	1,399 (13)	7,885 (19)	16,203 (4)	**
**Myocardial infarction, *N* (%)**						
Cases	1,899 (29)	3,921 (29)	276 (23)	1,516 (34)	5,855 (40)	**
Controls	4,396 (7)	20,217 (6)	340 (3)	1,776 (24)	21,553 (6)	**
**Heart failure, *N* (%)**						
Cases	2,518 (39)	5,554 (41)	537 (44)	3,135 (47)	2,585 (17)	**
Controls	2,438 (4)	9,388 (3)	221 (2)	1 (0)	2,392 (1)	**
**Valvular heart disease** ** **, *N* (%)**						
Cases	1,232 (19)	945 (7)	526 (43)	2,214 (33)	2,511 (17)	**
Controls	1,989 (3)	1,502 (0)	657 (6)	518 (1)	3,061 (1)	**
**Not diagnosed with hypertension, T2D, MI, HF, or valve disease, *N* (%)**						
Cases	548 (8)	6,135 (46)	137 (11)	379 (6)	2,310 (16)	**

T2D, type 2 diabetes; MI, myocardial infarction; HF, heart failure.

* Non-AF phenotypic information not complete. Valve disease based on aortic stenosis only.

** Please see christophersen et al, NG 2017 (PMID: 28416818) for details.

### Reverse direction analysis

**Figure 5:**
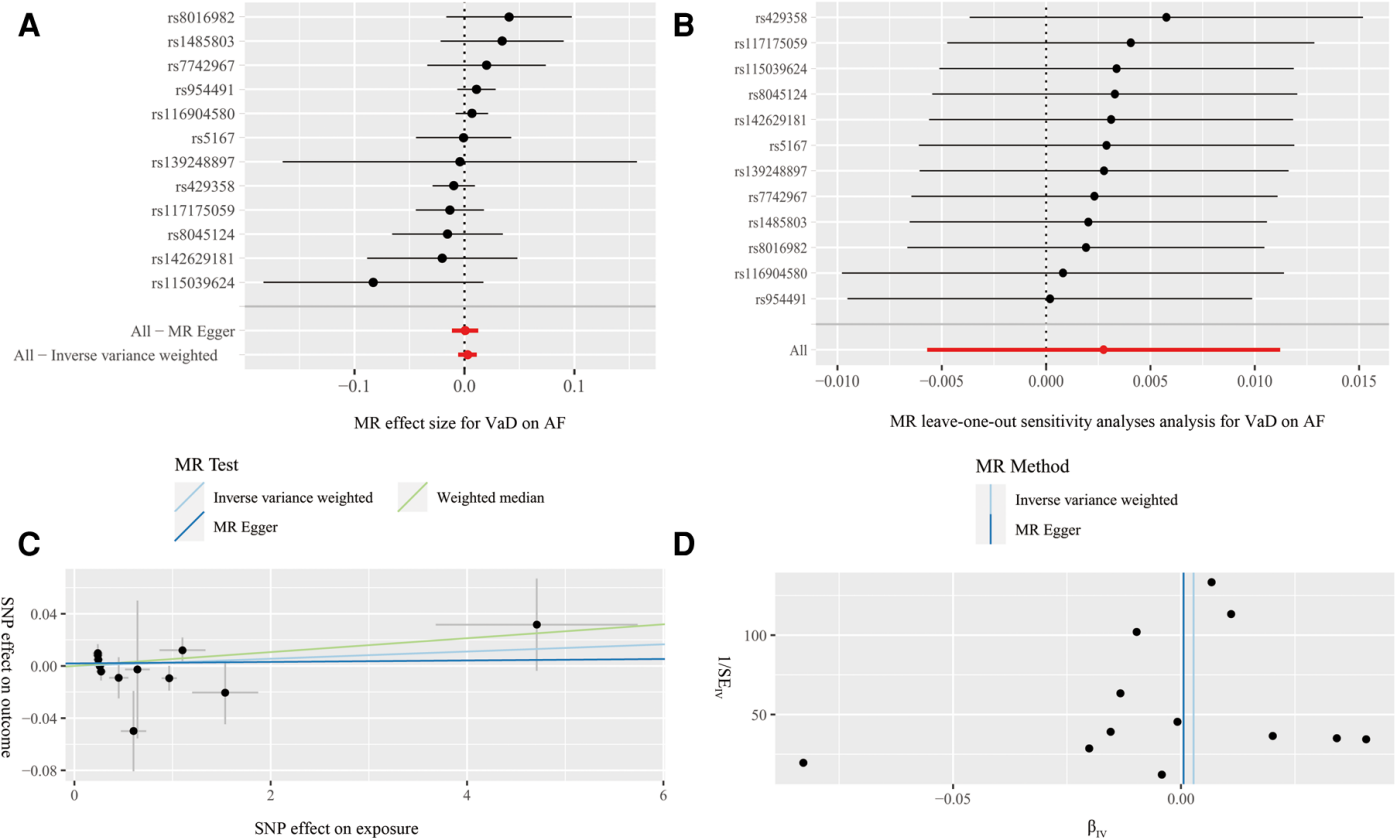
Forest plot (**A**), leave-one-out analysis (**B**), scatter plot (**C**) and funnel plot (**D)** of the effect of vascular dementia on atrial fibrillation (AF). Figure **A** presents the MR estimated effect sizes for AF. Figure **B** shows the changes in MR estimates after excluding each individual SNP. The three lines in Figure **C** illustrate the estimated effect sizes by three MR methods (IVW, MR-Egger and WM). The intercept of MR-Egger suggests no significant heterogeneity. Figure **D** shows that the funnel plot is symmetric and the MR result is robust.

We additionally performed a reverse MR study using VaD as exposure and AF as outcome to assess whether reverse causality exists. The flow of the reverse MR is shown in Figure 1. The results showed no significant causal relationship between VaD and AF risk using the IVW (OR = 1.00, 95%CI = 0.99–1.01, *P* = 0.52), MR-Egger (OR = 1.01, 95%CI = 0.99–1.02, *P* = 0.38), and WM (OR = 1.00, 95%CI = 0.99–1.01, *P* = 0.93) methods (Figure 5A and Table 2). In addition, cochran's Q statistic did not suggest significant heterogeneity (*P *= 0.45) (Table 2). The leave-one-out analysis did not find that our MR estimates were strongly affected by individual SNP (Figure 5B). The intercept of MR-Egger was 0.0019 (*P *= 0.61), which showed no significant horizontal pleiotropy (Figure 5C and Table 2). The funnel plot also showed that our results were reliable (Figure 5D).

## Discussion

We applied two-sample MR methods based on large-scale GWAS databases to investigate the causal genetic relationship between AF and VaD. It was demonstrated that there was no causal relationship between AF and the risk of developing VaD. Besides, no indication supports that VaD was causally related to AF risk.

Up to now, several observational studies have shown a significant relationship between AF and dementia (29–33). The prestigious Rotterdam Prospective Study, which recruited 81,483 participants for 20 years of follow-up, found that AF was significantly associated with all-cause dementia (OR = 1.33,95% CI = 1.02–1.73), and that this relationship was independent of stroke (32). In addition, a large meta-analysis by Papanastasiou et al. that included 43 observational studies yielded a similar conclusion (OR = 1.6,95% CI = 1.3–2.1). The subgroup analysis also confirmed that AF was related to a higher risk of both AD and VaD (11). However, there were some limitations in the previous meta-analysis, including confounding factors, reverse causality, inconsistent diagnostic criteria, and significant heterogeneity. Notably, The MR research by Pan et al. showed no causal relationship between genetically predicted AF and the risk of AD development (34). VaD is the second leading cause of dementia after AD (1). Currently, its causality with AF remains unknown in the field of genetic prediction.

It should be interpreted with caution that AF did not show a significant genetic causal relationship to VaD. One possible explanation is that VaD and AF share many common risk factors, such as hypertension, diabetes, hypercholesterolemia, arterial blockage and heart failure, and other risk factors may interfere with the relationship between AF and VaD risk in the real world (35, 36). A study involving 135 patients reported that AF, hypertension, and angina were associated with higher rates of cognitive dysfunction (37). Yaffe et al. discovered that lower cognitive function was associated with higher cumulative exposure to hyperglycemia, hypertension, and hypercholesterolemia in 3,381 patients (38). Regrettably, these studies did not adequately exclude the common risk factors, and the subgroup analysis of AF and VaD were not conducted in some studies. Importantly, our MR study only explored the genetic causality of AF and VaD. (i.e., the presence or absence of AF and VaD). Certain studies suggest that the occurrence of VaD may be related to the type and pattern of AF. Chen et al. found that cognitive function scores were generally lower in persistent AF (monitored by a 14-day ECG patch assessment) compared to paroxysmal AF based on the large prospective US cohort study based on the Atherosclerosis Risk in Communities (ARIC) (39). A sizable cohort study reported that patients with persistent AF showed lower cerebral blood flow and more severe poor brain perfusion than patients with paroxysmal AF (40). In terms of inflammation, a growing body of studies found higher inflammatory markers in patients with persistent AF compared to the paroxysmal AF population (41–43). A retrospective examination of 619 patients with cardiogenic stroke by Deguchi et al. revealed that individuals with persistent AF had worse prognoses, more severe neurological impairments, and a higher frequency of significant artery occlusions (44).

Although our MR study did not find significant genetic causal relationships between AF and VaD. However, we still have reason to believe that there are some other potential factors that we have not yet focused on or observed that contribute to this relationship in the real world. These potential factors might be common risk factors interference, the cumulative effect of persistent episodes of AF, and inherent limitations in observational studies. Future studies could be considered for in-depth optimization in this area.

Genetic and environmental factors often combine to cause disease. Future studies could focus on the interaction between environmental factors (e.g., lifestyle, dietary habits.) and genetic factors to better understand the relationship between AF and VaD. Recent observational studies suggest that oral anticoagulants and statins may protect cognitive function in AF patients (45–47). And future studies could further investigate the mechanisms and therapeutic effects of these drugs in preventing the development of VaD in patients with AF, which would provide new treatment options for clinical practice. In addition, cardioversion or radiofrequency ablation therapy may improve cognitive function in patients with AF, which may help in the clinical management of these patients (48).

To our knowledge, this is the first study to evaluate the causal relationships between AF and VaD using MR methods. Our study has many strengths. The first is the MR design, Genetic variation in genomic regions has larger sample sizes and is less prone to potentially unmeasurable confounders than traditional observational studies. Moreover, reverse causality can be prevented because genetic variants are assigned at the time of conception.

Our study has some limitations. Firstly, epigenetic issues (such as DNA methylation, non-coding RNA regulation and chromatin remodeling) in MR studies are challenging to avoid. Secondly, the winner's curse may overestimate the genetic relationships between AF and VaD. Thus, we used three MR methods to evaluate MR estimates to avoid this bias. These methods yielded consistent results, which suggests that the winner's curse in our MR analysis is relatively small. Fourthly, we were unable to further calculate the population overlap rate as the FinnGen did not provide details of VaD cohorts. Thirdly, most of the people in these datasets were of European ancestry, which restricts the application of our findings to populations with more varied ethnic backgrounds. Finally, we could not obtain complete demographic and clinical information, limiting us from conducting additional subgroup analysis.

## Conclusion

In conclusion, our MR study does not support a genetic causality between AF and VaD risk. Also, there was no causal relationships between VaD and AF risk. Updated MR studies will be needed when more valid statistical approaches are accessible to generate less biased results, or more extensive GWAS databases are available to obtain more accurate MR estimates. Secondly, further mechanistic studies and large-sample, multicenter, and follow-up studies are also warranted to validate our results in the real world. MR will need further to analyze other types of dementia and all-cause dementia when the GAWS database becomes more extensive. Thirdly, it is also important to emphasize that our MR analysis only investigates the genetic relationships, but other types of causal relationships cannot be revealed.

## Data Availability

The original contributions presented in the study are included in the article/**Supplementary Material**, further inquiries can be directed to the corresponding author/s.
