# Supplementary material for: No causal genetic relationships between atrial fibrillation and vascular dementia: A bidirectional Mendelian randomization study
Source: Front Cardiovasc Med. 2023 Jun 30;10:1071574. doi: 10.3389/fcvm.2023.1071574 (PMC10347408; doi:10.3389/fcvm.2023.1071574)
Supplement: Supplementary file 1 [file Table3.docx]

**Supplementary Material S1 Formula for calculating *F* statistic**

R^2^ = 2  × EAF × (1 − EAF) × (**β**)^2^

*F* statistic= R^2^ × (N − 2) / (1 − R^2^)

NOTE: R^2^(The variance):the proportion of exposed variability explained by individual genetic instrument; EAF: the effect allele frequency; **β**: the estimated effect of SNP; Ν: the sample size of the GWAS.

**Table S1.** Characteristics of instrumental variables for [atrial fibrillation](http://www.baidu.com/link?url=m46YKWzsYnQ6j0UXpVWRXq3MxSXA6Au5y7VCGtgEfsQXOVApH6rTxDuDGkaN6iP6b_bPHv3YruU-jnf5XR1_0QDYHitHDFUv9PjuLqVFPG2gw8ZN4adwxAqsvTlX-TeH)

| **SNP** | **Gene** | **Samplesize** | **EA** | **OA** | **β** | **SE** | **EAF** | ***P* value** | **R^2^** | **F-static** |
| --- | --- | --- | --- | --- | --- | --- | --- | --- | --- | --- |
| rs10753933 | PPFIA4 | 1030836 | G | T | -0.0609 | 0.0067 | 0.5518 | 9.84E-20 | 0.0018 | 1887.60 |
| rs11264280 | KCNN3 | 1030836 | T | C | 0.1347 | 0.0071 | 0.3330 | 3.07E-79 | 0.0081 | 8241.56 |
| rs4073778 | CASQ2 | 1030836 | A | C | 0.0486 | 0.0067 | 0.5639 | 4.96E-13 | 0.0012 | 1196.12 |
| rs72694603 | KCND3 | 1030836 | T | C | -0.0553 | 0.0072 | 0.3147 | 2.26E-14 | 0.0013 | 1357.92 |
| rs577676 | RP1-79C4.1 | 1030836 | T | C | -0.0923 | 0.0067 | 0.4383 | 1.62E-43 | 0.0042 | 4305.98 |
| rs79187193 | RP11-433J22.3 | 1030836 | A | G | -0.1162 | 0.0153 | 0.0569 | 3.15E-14 | 0.0014 | 1491.66 |
| rs72700114 | LINC01681 | 1030836 | C | G | 0.2021 | 0.0130 | 0.0756 | 3.29E-54 | 0.0057 | 5851.22 |
| rs6665642 | KCNN3 | 1030836 | T | C | -0.0620 | 0.0112 | 0.1176 | 3.06E-08 | 0.0008 | 821.73 |
| rs146518726 | MIR4421 | 1030836 | A | G | 0.1605 | 0.0207 | 0.0328 | 8.27E-15 | 0.0016 | 1682.09 |
| rs74832855 | LINC01681 | 1030836 | G | A | 0.1216 | 0.0180 | 0.0369 | 1.43E-11 | 0.0011 | 1082.25 |
| rs7529220 | RP11-26H16.4 | 1030836 | C | T | 0.0621 | 0.0098 | 0.8469 | 1.98E-10 | 0.0010 | 1029.85 |
| rs2885697 | SCMH1 | 1030836 | T | G | -0.0439 | 0.0070 | 0.6482 | 2.88E-10 | 0.0009 | 905.25 |
| rs6546620 | KIF3C | 1030836 | C | T | 0.0602 | 0.0086 | 0.7999 | 3.19E-12 | 0.0012 | 1194.51 |
| rs28387148 | GYPC | 1030836 | T | C | 0.0741 | 0.0113 | 0.1051 | 6.25E-11 | 0.0010 | 1063.61 |
| rs6747542 | GMCL1 | 1030836 | C | T | -0.0554 | 0.0067 | 0.4642 | 1.10E-16 | 0.0015 | 1571.38 |
| rs7574892 | WIPF1 | 1030836 | A | G | 0.0552 | 0.0067 | 0.4847 | 1.98E-16 | 0.0015 | 1566.64 |
| rs67969609 | TEX41 | 1030836 | G | C | 0.0711 | 0.0126 | 0.0710 | 1.71E-08 | 0.0007 | 686.98 |
| rs35544454 | ERBB4 | 1030836 | T | A | -0.0589 | 0.0087 | 0.1918 | 1.10E-11 | 0.0011 | 1107.51 |
| rs72926475 | SNORA19 | 1030836 | A | G | -0.0683 | 0.0102 | 0.1228 | 2.37E-11 | 0.0010 | 1034.95 |
| rs2288327 | TTN | 1030836 | G | A | 0.0919 | 0.0089 | 0.1564 | 7.26E-25 | 0.0022 | 2292.21 |
| rs56326533 | SPATS2L | 1030836 | C | T | 0.0685 | 0.0068 | 0.3919 | 6.28E-24 | 0.0022 | 2300.26 |
| rs4642101 | CAND2 | 1030836 | G | T | 0.0706 | 0.0069 | 0.6397 | 2.95E-24 | 0.0023 | 2363.03 |
| rs6771054 | EPHA3 | 1030836 | C | T | -0.0457 | 0.0068 | 0.4035 | 2.42E-11 | 0.0010 | 1035.30 |
| rs7612445 | GNB4 | 1030836 | T | G | 0.0493 | 0.0084 | 0.1879 | 4.81E-09 | 0.0007 | 764.06 |
| rs62254082 | FRMD4B | 1030836 | C | T | 0.0404 | 0.0070 | 0.3865 | 6.34E-09 | 0.0008 | 797.28 |
| rs1278493 | PPP2R3A | 1030836 | A | G | -0.0389 | 0.0068 | 0.5645 | 8.77E-09 | 0.0007 | 766.38 |
| rs34080181 | LRIG1 | 1030836 | A | G | -0.0446 | 0.0069 | 0.3790 | 1.28E-10 | 0.0009 | 964.30 |
| rs73041705 | THRB | 1030836 | C | T | -0.0443 | 0.0073 | 0.2985 | 1.55E-09 | 0.0008 | 846.53 |
| rs60902112 | XXYLT1 | 1030836 | T | C | 0.0445 | 0.0079 | 0.2262 | 1.72E-08 | 0.0007 | 714.10 |
| rs6790396 | SCN10A | 1030836 | G | C | 0.0627 | 0.0068 | 0.5959 | 2.40E-20 | 0.0019 | 1948.02 |
| rs10804493 | PHLDB2 | 1030836 | A | G | 0.0558 | 0.0070 | 0.6505 | 1.63E-15 | 0.0014 | 1457.36 |
| rs6838973 | RP11-119H12.3 | 1030836 | T | C | -0.1514 | 0.0067 | 0.4406 | 1.03E-111 | 0.0113 | 11516.02 |
| rs74500426 | RN7SKP13 | 1030836 | T | G | -0.0921 | 0.0127 | 0.0764 | 4.29E-13 | 0.0012 | 1232.52 |
| rs1906615 | RP11-119H12.3 | 1030836 | T | G | 0.3658 | 0.0081 | 0.1991 | 1.00E-200 | 0.0427 | 42112.92 |
| rs2739197 | NKX2-5 | 1030836 | G | C | 0.1160 | 0.0086 | 0.2458 | 3.20E-41 | 0.0050 | 5117.18 |
| rs10213171 | ARHGAP10 | 1030836 | G | C | 0.0910 | 0.0134 | 0.0609 | 1.32E-11 | 0.0009 | 975.48 |
| rs10520260 | HAND2 | 1030836 | G | A | -0.0457 | 0.0073 | 0.3214 | 3.36E-10 | 0.0009 | 938.24 |
| rs17171711 | FAM13B | 1030836 | T | C | 0.1086 | 0.0087 | 0.1775 | 1.95E-35 | 0.0034 | 3537.65 |
| rs6580277 | NR3C1 | 1030836 | G | A | 0.0670 | 0.0079 | 0.2369 | 1.64E-17 | 0.0016 | 1670.36 |
| rs337705 | KCNN2 | 1030836 | G | T | 0.0564 | 0.0068 | 0.3749 | 1.63E-16 | 0.0015 | 1534.60 |
| rs62377206 | SLIT3 | 1030836 | A | G | 0.0846 | 0.0147 | 0.0554 | 8.21E-09 | 0.0007 | 771.60 |
| rs6882776 | NKX2-5 | 1030836 | A | G | -0.0711 | 0.0074 | 0.2835 | 9.64E-22 | 0.0021 | 2112.68 |
| rs6596717 | RP11-231G15.1 | 1030836 | A | C | -0.0404 | 0.0068 | 0.6049 | 3.00E-09 | 0.0008 | 803.59 |
| rs10520002 | FBN2 | 1030836 | A | G | 0.0626 | 0.0113 | 0.0988 | 2.85E-08 | 0.0007 | 718.86 |
| rs72966339 | TRMT11 | 1030836 | T | C | -0.0616 | 0.0069 | 0.3679 | 7.42E-19 | 0.0018 | 1816.05 |
| rs2031522 | RP11-374I15.1 | 1030836 | G | A | -0.0436 | 0.0068 | 0.3764 | 1.47E-10 | 0.0009 | 919.09 |
| rs34969716 | KDM1B | 1030836 | A | G | 0.0702 | 0.0078 | 0.3051 | 1.60E-19 | 0.0021 | 2149.56 |
| rs3176326 | CDKN1A | 1030836 | A | G | -0.0626 | 0.0085 | 0.1982 | 1.42E-13 | 0.0012 | 1282.32 |
| rs73366713 | ATXN1 | 1030836 | A | G | -0.1035 | 0.0099 | 0.1396 | 1.53E-25 | 0.0026 | 2645.86 |
| rs4946333 | SLC35F1 | 1030836 | G | A | 0.0639 | 0.0066 | 0.4897 | 5.47E-22 | 0.0020 | 2099.37 |
| rs117984853 | UST | 1030836 | T | G | 0.1228 | 0.0120 | 0.1013 | 1.34E-24 | 0.0027 | 2822.58 |
| rs55734480 | DGKB | 1030836 | A | G | 0.0548 | 0.0078 | 0.2494 | 2.20E-12 | 0.0011 | 1157.70 |
| rs55985730 | OPN1SW | 1030836 | G | T | 0.0867 | 0.0149 | 0.0600 | 5.24E-09 | 0.0008 | 873.31 |
| rs74910854 | GTF2I | 1030836 | G | A | 0.0900 | 0.0164 | 0.0693 | 4.31E-08 | 0.0010 | 1075.95 |
| rs7789146 | KCNH2 | 1030836 | A | G | -0.0584 | 0.0087 | 0.1787 | 2.12E-11 | 0.0010 | 1030.95 |
| rs6462079 | CREB5 | 1030836 | A | G | 0.0466 | 0.0076 | 0.7208 | 8.79E-10 | 0.0009 | 900.20 |
| rs11773845 | CAV1 | 1030836 | A | C | 0.1054 | 0.0067 | 0.5856 | 2.39E-55 | 0.0054 | 5528.06 |
| rs62521286 | FBXO32 | 1030836 | G | A | 0.1202 | 0.0135 | 0.0663 | 4.50E-19 | 0.0018 | 1840.65 |
| rs3943207 | XPO7 | 1030836 | T | G | -0.0638 | 0.0103 | 0.1167 | 6.92E-10 | 0.0008 | 864.32 |
| rs6994744 | PTK2 | 1030836 | C | A | 0.0405 | 0.0066 | 0.4954 | 1.09E-09 | 0.0008 | 844.65 |
| rs35963991 | GATA4 | 1030836 | T | G | 0.0525 | 0.0095 | 0.1494 | 2.80E-08 | 0.0007 | 721.62 |
| rs7508 | ASAH1 | 1030836 | A | G | 0.0711 | 0.0075 | 0.7109 | 1.69E-21 | 0.0021 | 2137.52 |
| rs10821415 | C9orf3 | 1030836 | A | C | 0.0821 | 0.0067 | 0.4132 | 2.92E-34 | 0.0033 | 3358.41 |
| rs2274115 | LHX3 | 1030836 | G | A | 0.0487 | 0.0076 | 0.7003 | 1.69E-10 | 0.0010 | 1025.21 |
| rs10458662 | LRMDA | 1030836 | G | T | 0.0544 | 0.0088 | 0.1722 | 6.93E-10 | 0.0008 | 868.98 |
| rs11598047 | NEURL1 | 1030836 | G | A | 0.1537 | 0.0090 | 0.1621 | 8.95E-66 | 0.0064 | 6572.73 |
| rs34936990 | SH3PXD2A | 1030836 | A | G | 0.1294 | 0.0101 | 0.1207 | 2.95E-37 | 0.0036 | 3650.78 |
| rs4757877 | NAV2 | 1030836 | G | A | -0.0723 | 0.0078 | 0.7552 | 2.93E-20 | 0.0019 | 1988.51 |
| rs4935786 | SORL1 | 1030836 | A | T | -0.0463 | 0.0079 | 0.7327 | 4.85E-09 | 0.0008 | 864.85 |
| rs76097649 | KCNJ5 | 1030836 | A | G | 0.1151 | 0.0124 | 0.0933 | 1.26E-20 | 0.0022 | 2305.37 |
| rs10842383 | RP11-615I16.1 | 1030836 | T | C | -0.0988 | 0.0095 | 0.1478 | 2.88E-25 | 0.0025 | 2528.60 |
| rs71454237 | LRRC10 | 1030836 | A | G | -0.0620 | 0.0084 | 0.2090 | 1.78E-13 | 0.0013 | 1308.50 |
| rs12426679 | RP11-114H23.1 | 1030836 | T | C | -0.0391 | 0.0067 | 0.5278 | 4.95E-09 | 0.0008 | 784.94 |
| rs6560886 | FBRSL1 | 1030836 | C | T | 0.0510 | 0.0090 | 0.7884 | 1.49E-08 | 0.0009 | 893.81 |
| rs883079 | TBX5 | 1030836 | T | C | 0.0981 | 0.0074 | 0.7074 | 2.84E-40 | 0.0040 | 4090.37 |
| rs17380837 | SSPN | 1030836 | T | C | -0.0501 | 0.0072 | 0.3070 | 4.80E-12 | 0.0011 | 1099.77 |
| rs2860482 | NACA | 1030836 | C | A | -0.0540 | 0.0076 | 0.7260 | 1.21E-12 | 0.0012 | 1194.51 |
| rs775498 | BEST3 | 1030836 | G | A | 0.0423 | 0.0074 | 0.2798 | 1.05E-08 | 0.0007 | 742.83 |
| rs10773657 | HIP1R | 1030836 | A | C | -0.0575 | 0.0103 | 0.8620 | 2.54E-08 | 0.0008 | 810.21 |
| rs35569628 | CUL4A | 1030836 | C | T | -0.0452 | 0.0080 | 0.2230 | 1.38E-08 | 0.0007 | 729.31 |
| rs9506925 | SNORD36 | 1030836 | T | C | 0.0449 | 0.0075 | 0.2669 | 2.72E-09 | 0.0008 | 812.61 |
| rs74884082 | DPF3 | 1030836 | T | C | -0.0493 | 0.0078 | 0.2495 | 3.48E-10 | 0.0009 | 937.43 |
| rs4587869 | AKAP6 | 1030836 | C | G | 0.0716 | 0.0077 | 0.2849 | 1.19E-20 | 0.0021 | 2148.80 |
| rs2738413 | SYNE2 | 1030836 | G | A | -0.0778 | 0.0067 | 0.5049 | 2.55E-31 | 0.0030 | 3110.00 |
| rs10141892 | CFL2 | 1030836 | C | T | -0.0452 | 0.0068 | 0.5833 | 2.95E-11 | 0.0010 | 1022.77 |
| rs28631169 | MYH7 | 1030836 | T | C | 0.0522 | 0.0084 | 0.1982 | 5.35E-10 | 0.0009 | 891.98 |
| rs7172038 | HCN4 | 1030836 | G | T | 0.1120 | 0.0089 | 0.1597 | 4.78E-36 | 0.0034 | 3458.83 |
| rs4965430 | IGF1R | 1030836 | G | C | -0.0441 | 0.0069 | 0.6136 | 1.26E-10 | 0.0009 | 949.77 |
| rs2359171 | ZFHX3 | 1030836 | A | T | 0.1746 | 0.0086 | 0.1760 | 4.65E-91 | 0.0088 | 9034.20 |
| rs77316573 | PGP | 1030836 | T | C | 0.0529 | 0.0089 | 0.1991 | 3.26E-09 | 0.0009 | 919.16 |
| rs140185678 | RPL3L | 1030836 | A | G | 0.1659 | 0.0218 | 0.0351 | 2.43E-14 | 0.0019 | 1918.19 |
| rs1563304 | WNT3 | 1030836 | T | C | 0.0644 | 0.0092 | 0.1780 | 2.56E-12 | 0.0012 | 1249.55 |
| rs4252627 | ERBB2 | 1030836 | T | C | -0.0415 | 0.0071 | 0.6679 | 5.63E-09 | 0.0008 | 786.98 |
| rs72811294 | MYOCD | 1030836 | C | G | -0.0720 | 0.0106 | 0.1131 | 9.67E-12 | 0.0010 | 1070.95 |
| rs7225165 | YWHAE | 1030836 | A | G | -0.0655 | 0.0111 | 0.1133 | 3.20E-09 | 0.0009 | 887.84 |
| rs7224711 | CYTH1 | 1030836 | T | C | -0.0365 | 0.0066 | 0.5222 | 3.72E-08 | 0.0007 | 684.86 |
| rs9953366 | SMAD7 | 1030836 | C | T | 0.0490 | 0.0073 | 0.6631 | 1.82E-11 | 0.0011 | 1104.65 |
| rs8088085 | MEX3C | 1030836 | C | A | -0.0365 | 0.0067 | 0.4646 | 4.79E-08 | 0.0007 | 682.77 |
| rs2834618 | LINC01426 | 1030836 | G | T | -0.0944 | 0.0112 | 0.1056 | 3.41E-17 | 0.0017 | 1732.31 |
| rs464901 | TUBA8 | 1030836 | C | T | -0.0508 | 0.0072 | 0.3353 | 1.53E-12 | 0.0012 | 1184.42 |
| rs133885 | MYO18B | 1030836 | A | G | 0.0405 | 0.0068 | 0.4377 | 2.22E-09 | 0.0008 | 831.62 |

SNP:single nucleotide polymorphisms; EA: effect allele; OA: other allele; EAF: effect allele frequency; SE, standard error

**Table S2.** Characteristics of instrumental variables for Vascular dementia

| **SNP** | **Gene** | **Samplesize** | **EA** | **OA** | **β** | **SE** | **EAF** | ***P* value** | **R^2^** | **F-static** |
| --- | --- | --- | --- | --- | --- | --- | --- | --- | --- | --- |
| rs142629181 | HFM1 | 212,389 | T | G | 0.4520 | 0.0978 | 0.0700 | 9.84E-20 | 0.0266 | 5806.33 |
| rs954491 | LRRTM4 | 212,390 | G | T | 1.1015 | 0.2340 | 0.0144 | 3.07E-79 | 0.0345 | 7580.90 |
| rs115039624 | RP11-576N17.4 | 212,391 | G | A | 0.6026 | 0.1287 | 0.0419 | 4.96E-13 | 0.0292 | 6379.66 |
| rs7742967 | RNU7-66P | 212,392 | C | A | 0.2433 | 0.0494 | 0.4656 | 2.26E-14 | 0.0295 | 6446.34 |
| rs117175059 | ANKMY2 | 212,393 | T | C | 1.5364 | 0.3362 | 0.0074 | 1.62E-43 | 0.0345 | 7580.96 |
| rs116904580 | RN7SL167P | 212,394 | A | G | 4.7099 | 1.0284 | 0.0013 | 3.15E-14 | 0.0559 | 12580.37 |
| rs8016982 | DYNLL1P1 | 212,395 | T | C | 0.2388 | 0.0507 | 0.6178 | 3.29E-54 | 0.0269 | 5878.05 |
| rs8045124 | CRAMP1 | 212,396 | C | T | -0.2722 | 0.0497 | 0.4807 | 3.06E-08 | 0.0370 | 8158.51 |
| rs1485803 | CHST9 | 212,397 | A | G | 0.2396 | 0.0497 | 0.4663 | 8.27E-15 | 0.0286 | 6247.42 |
| rs429358 | APOE | 212,398 | C | T | 0.9663 | 0.0769 | 0.1791 | 1.43E-11 | 0.2746 | 80386.96 |
| rs139248897 | IRF2BP1 | 212,399 | A | G | 0.6423 | 0.1257 | 0.0474 | 1.98E-10 | 0.0372 | 8215.83 |
| rs5167 | APOC4 | 212,400 | G | T | 0.2587 | 0.0506 | 0.4134 | 2.88E-10 | 0.0325 | 7125.52 |

SNP:single nucleotide polymorphisms; EA: effect allele; OA: other allele; EAF: effect allele frequency; SE, standard error
